# Martensitic Transformation Mechanism In Situ Observation for the Simulated Coarse-Grained Heat-Affected Zone of DP1180 Steel

**DOI:** 10.3390/ma18122721

**Published:** 2025-06-10

**Authors:** Wenjuan Li, Jinfeng Wang, Wenchao Su, Zhiyuan Wei, Jiaxin Wu, Xiaofei Xu, Jiaan Wei

**Affiliations:** 1 School of Automotive Materials, Hubei University of Automotive Technology, Shiyan 442002, China; 2 State Key Laboratory of Advanced Welding and Joining, Harbin Institute of Technology, Harbin 150001, China

**Keywords:** dual phase steel, coarse grain heat-affected zone, phase transition behavior, in situ observation, transformation kinetics

## Abstract

The martensitic transformation mechanism in the heat-affected zone of DP1180 steel plays a decisive role in the strength of welded joints. In this work, the nucleation and growth kinetics of martensite laths in the coarse grain heat-affected zone (CGHAZ) are analyzed by a high-temperature laser scanning confocal microscope (LSCM). The grain distribution and stress distribution of the samples after in situ observation are analyzed by electron backscatter diffraction (EBSD). The results reveal that austenite grain growth is realized by continuous grain boundary annexation and grain boundary migration of small grains by large grains during the heating process. Seven growth modes of CGHAZ martensitic laths under laser welding conditions are proposed. Additionally, the end growth of martensitic laths is mostly attributed to the collision with grain boundaries or other laths to form a complex interlocking structure. The results of this study could provide important data support for the development of dual-phase steel materials and improvement of welding quality.

## 1. Introduction

The primary task of the modern automobile industry is to design and manufacture automobiles with high safety and light weight [1,2]. To reduce the weight of automobiles and improve safety, the steel industry has quickly turned its attention to higher strength steel, known as advanced high-strength steel (AHSS) [3,4]. Dual-phase steel (DP Steel) in AHSS has received widespread attention due to its excellent mechanical properties and good processability [5]. DP1180 steel is a typical high-strength dual-phase steel. The phase transformation behavior of the heat-affected zone (HAZ) of DP1180 is quite complex during laser welding. In particular, a large grain size is generated in the coarse-grained heat-affected zone (CGHAZ), resulting in a serious loss of toughness in the welded joint [6,7,8,9].

Conventional material characterization techniques, such as scanning electron microscopy (SEM) and transmission electron microscopy (TEM), can provide information on the microstructure of materials [10,11]. However, these techniques cannot capture the dynamic changes in the microstructure during the welding process in real time, which limits the understanding of the phase transformation mechanism of the welding HAZ. The development of high-temperature laser scanning confocal microscope (LSCM) technology has broken this limitation. LSCM technology can not only provide high-resolution three-dimensional images but also capture microstructure changes in materials during heating and cooling in real time. The phase transformation process of material under a simulated welding thermal cycle has been observed intuitively, including the formation of austenite, the transformation of martensite, and the formation of possible precipitated phases [10,11,12,13,14].

Many researchers have used the LSCM to study the grain growth and phase transition behavior of materials. Hu et al. [15] investigated the phase transformation behavior in Fe-C-Mn-Si super bainite steel. Mao et al. [10,16] analyzed the microstructure transformation of weld metals with different Ni contents at two cooling rates and the kinetic process of bainite nucleation and growth in reheated weld metals. Several growth behaviors and nucleation characteristics of bainite laths were proposed based on the phase transformation-induced relief phenomenon. Li et al. [17] studied the martensitic transformation behavior in Nm500 wear-resistant steel at different quenching temperatures. The martensitic laths appear explosively with the continuous decrease in temperature during the whole martensitic transformation process. Most of the martensite passes through the grains after nucleation and growth of prior austenite grain boundaries (PAGBs). A small portion of the martensitic laths ends up growing after encountering other laths. Shen et al. [18] presented the phase transformation behavior of the HAZ, as well as the growth characteristics and phase transformation kinetics of laths, in three Cr-Mo ferritic heat-resistant steels, P11, P22, and P91, during continuous cooling. The results showed that bainite transformation was observed in the HAZ of P11 and P22 ferritic heat-resistant steels. Martensitic transformation was observed in the HAZ of P91 ferritic heat-resistant steel. Martensitic laths grow rapidly inside the grains through a non-diffusive shear mechanism during the phase transformation process. Yang et al. [19] studied the mechanism of austenite grain growth and martensitic transformation in 40Cr10Si2Mo steel. The results showed that the austenite grain size of 40Cr10Si2Mo steel increased with the increase in temperature and time during the isothermal process of 1800 s in the temperature range of 900 °C–1250 °C. Yuan et al. [20] analyzed the effect of heating rate on the microstructure evolution in a new Ti-Mo martensitic steel by in situ observation using the LSCM. The results showed that rapid heating promoted the refinement of austenite grains, and the coherent and incoherent relationship between (Ti, Mo, Fe) C particles and martensite matrix during rapid heating had a significant pinning effect on grain boundary migration. However, the martensitic transformation behavior and microstructure characteristics of the CGHAZ welded by DP1180 have not been reported yet.

This work aimed to dynamically analyze the martensitic transformation behavior of the CGHAZ in DP1180 steel during the simulated welding thermal cycle using the LSCM. The samples were characterized by EBSD after in situ observation. The underlying mechanisms of austenite grain growth, martensite lath nucleation and growth, and its phase transition kinetics were explored.

## 2. Experimental Procedures

**Figure 1:**
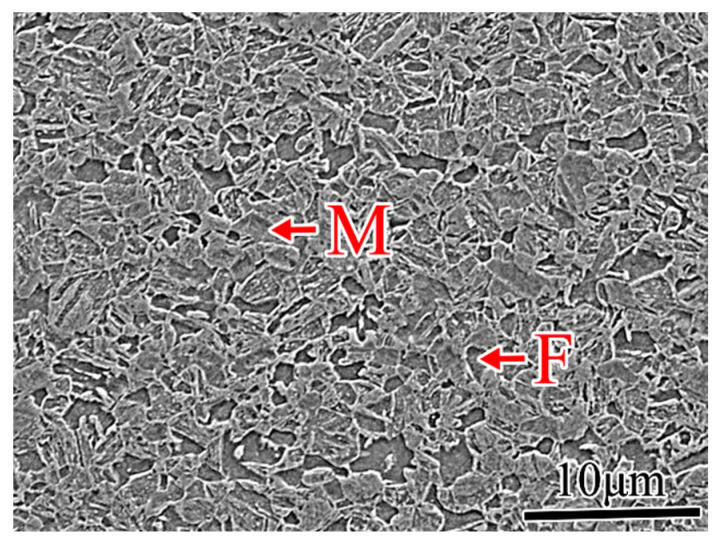
Microstructure of DP1180 steel.

The welding thermal simulation specimens were cut from the 1.2 mm thick steel sheet in the rolling direction. Its primary chemical composition is detailed in Table 1, while Table 2 outlines its basic mechanical properties. The room-temperature microstructure of DP1180 steel predominantly consists of martensite and ferrite, as illustrated in Figure 1.

**Figure 2:**
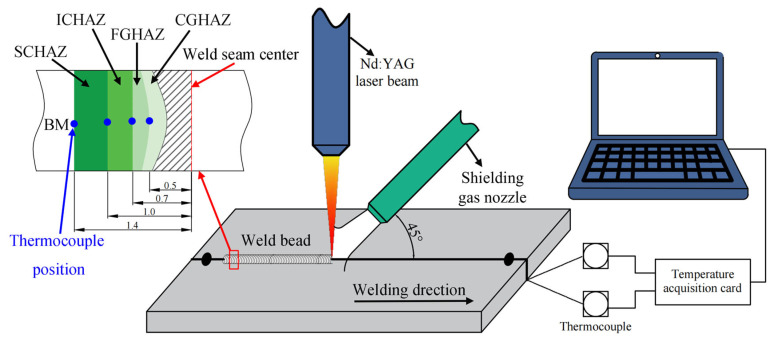
Schematic diagram of temperature test system.

**Figure 3:**
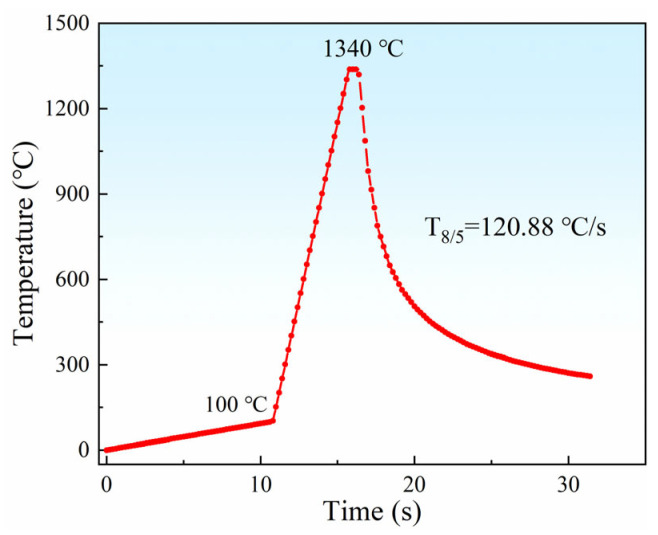
Welding thermal simulation curve of DP1180 steel.

Figure 2 shows a schematic diagram of the temperature test system. The DP1180 steel plate (size: 200 × 100 × 1.2 mm) is welded by a JK2003SM (GSI, London, UK) Nd: YAG laser (Wuhan, CHN). The process parameters are as follows: 2.6 kW laser power, 28 mm/s welding speed, and−1 mm defocusing amount. According to the experience of many experiments, it can be determined that the coarse-grained zone, fine-grained zone, inter-critical zone, and sub-critical zone are, respectively, 0.5, 0.7, 1.0 and 1.4 mm away from the center of weld pool. The thermal cycle curve of each region can be measured by arranging the K-type thermocouple above the position, and then the temperature change curve of the thermal simulation can be determined (see Figure 3).

**Figure 4:**
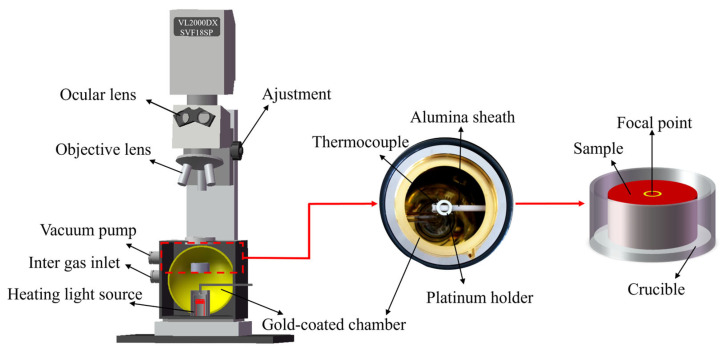
In situ observation equipment diagram.

**Figure 5:**
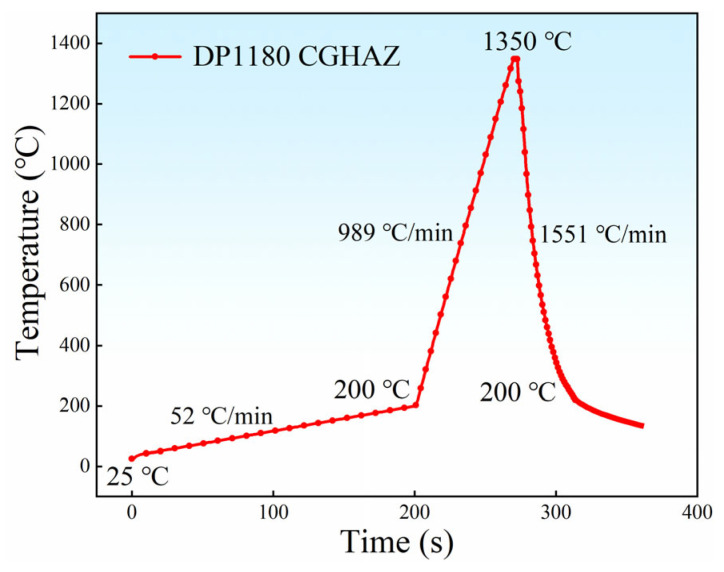
Welding thermal cycle curve of DP1180 steel.

The material is cut into a disk with a diameter of 7 mm and a thickness of 1.2 mm. In situ observation experiments are performed on a VL2000DX-SVF18SP ultra-high temperature laser confocal microscope (YONEKURA MFG Co., Ltd., Yokohama, Japan), as shown in Figure 4. Before the experiment, the upper and lower surfaces and circumferences of the sample are polished with sandpaper, until the metal luster is completely exposed. Then, the upper and lower surfaces of the sample are ground using 240 #, 800 #, 1200 #, and 1500 # sandpaper in turn, while ensuring that the two horizontal surfaces are parallel. Subsequently, the observed surface is mechanically polished with a gold velvet polishing cloth. In the experiment, the prepared samples are placed in a ceramic crucible with a diameter of 7.8 mm and a height of 3.5 mm and set on a Pt sample bracket. The samples are cleaned with high-purity Ar gas (99.99%) three times after sealing the heating furnace, which is then filled with Ar gas to prevent oxidation during the whole experiment. According to the above experimental results, Figure 5 shows the welding thermal cycle curve of DP1180 steel by the LSCM. First, it increases to 200 °C at 53 °C/min, and then rapidly increases to 1350 °C at 989 °C/min. Then, it rapidly decreases to 200 °C at a rate of 1551 °C/min, finally dropping to room temperature. The curve is specially used to reproduce the welding thermal cycle process of the CGHAZ under laser welding conditions. The charge-coupled device (CCD) camera in the LCSM captures real-time images at a rate of 20 frames per second during the cooling process. The in situ observation results of the whole experiment are recorded in the video file.

The previously mentioned in situ observed samples are subjected to conventional mechanical grinding and polishing, and the stress layer on the surface is eliminated by electrolytic polishing to obtain a flat surface without deformation damage. The electrolytic polishing solution used in this work is 5% perchloric acid–alcohol solution (volume fraction). The polishing temperature is room temperature, the polishing voltage is 20 V, and the polishing time is 30 s. Subsequently, crystallographic information is collected to fix the electrolytically polished sample on the Symmerfeld Apreo 2 s field emission electron microscope (OXIG, Abingdon, UK). In the experiment, the sample is tilted by 70°, the scanning step is 0.2 μm, and the acceleration voltage is 20 kV, with follow-up data processing using AZtecCrystal 2.1 software.

## 3. Experimental Results

### 3.1. Austenite Grain Growth Behavior

In this study, the sample is heated to 1350 °C to simulate the CGHAZ phase transformation behavior under laser welding conditions. In this section, the austenite growth behavior during heating is observed and analyzed.

**Figure 6:**
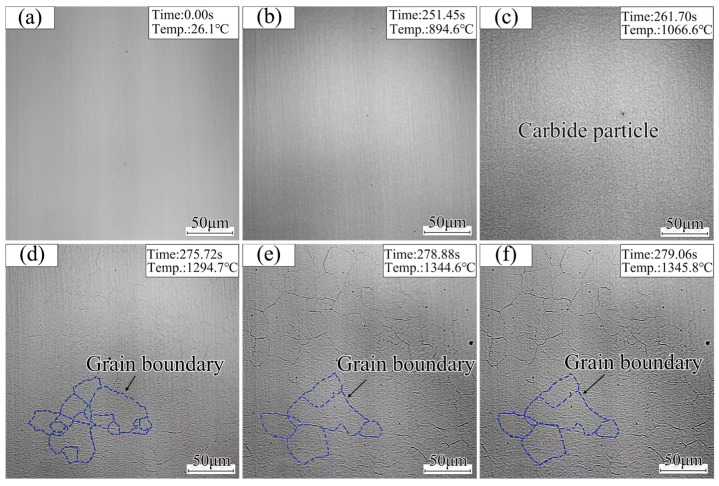
Austenite grain growth behavior LSCM image.

Figure 6a–f shows the in situ observation of the austenite grain growth behavior of DP1180 steel during heating. Austenite grain boundaries are blurred and difficult to observe at the beginning of the temperature rise in Figure 6a. The austenite grain boundary profile began to appear until the temperature reached 894.6 °C, as shown in Figure 6b. As the temperature increased to 1066.6 °C, a significant amount of carbide precipitated on the sample surface, as shown in Figure 6c. Subsequently, when the temperature reached about 1294.7 °C, the carbide particles were completely absorbed by the austenite grain boundary. At the same time, the austenite grain boundary contour became clear, the grains began to grow, and the growth rate increased greatly, as shown in Figure 6d. The austenite grain growth continued until 1344.6 °C, during which a small number of carbides precipitated on the grain boundaries (see Figure 6e). Beyond this temperature, the austenite grain size remained stable, and no further carbide precipitation was observed, as illustrated in Figure 6f.

The analysis shows that the grain growth process includes the merging of multiple small grains into large grains and the growth of large grains. The initial grains are usually fine and unevenly distributed, and the interface energy is high at the initial stage of austenite formation. From a thermodynamic perspective, the higher the interfacial energy, the more unstable the interface becomes. The system will spontaneously reduce the grain boundary area in order to lower the interfacial energy, which is typically achieved by the merging of multiple small grains into larger grains. The driving force for grain growth is primarily associated with the grain boundary curvature and the magnitude of the interfacial energy [21]. The greater the interfacial energy and the smaller the grain boundary curvature, the stronger the driving force and, consequently, the greater the tendency for grain growth, which means that the grain boundary is more likely to migrate.

### 3.2. Nucleation and Growth Mode of Martensitic Laths

**Figure 7:**
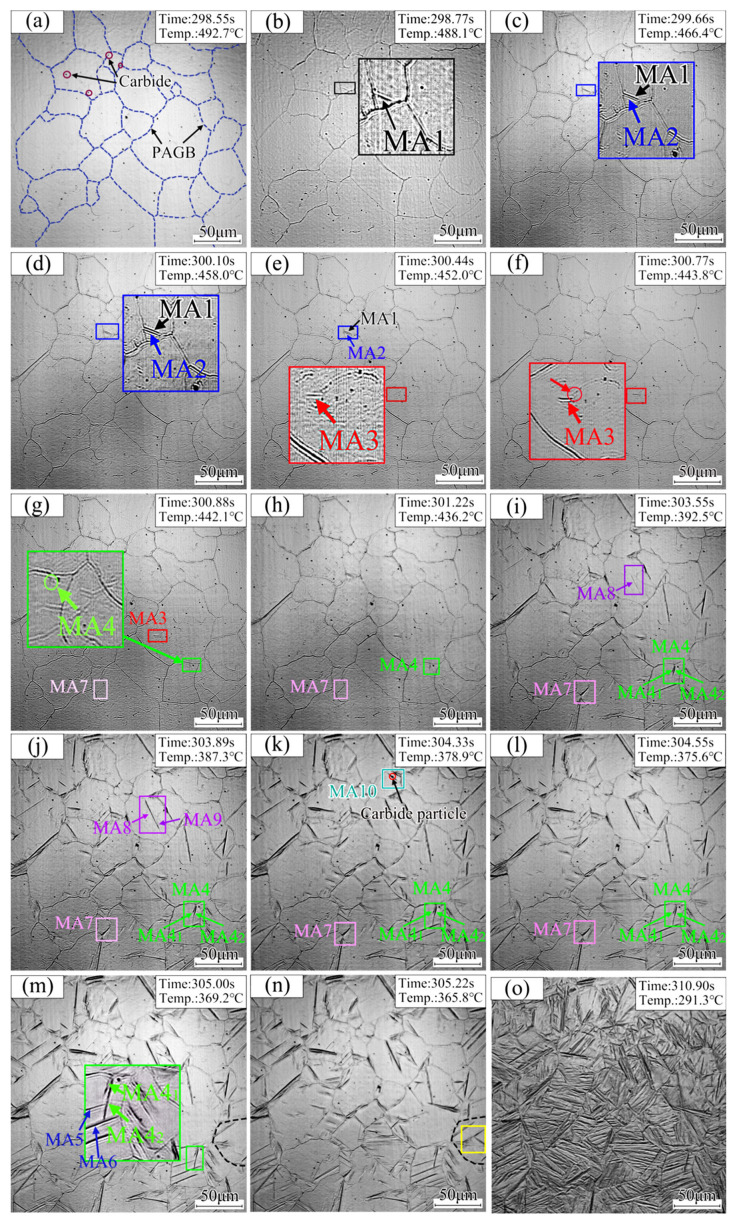
Evolution of martensitic lath growth at different positions during cooling.

**Figure 8:**
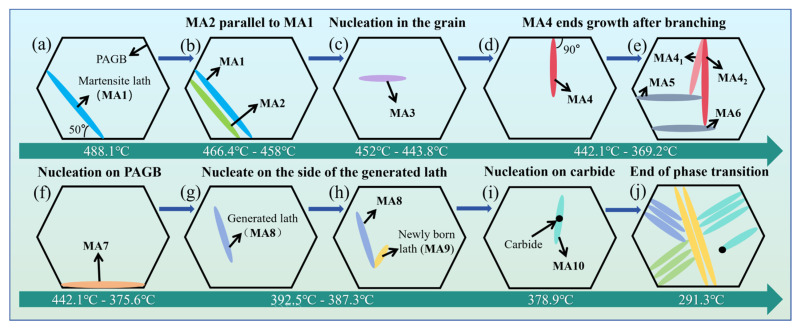
Model diagram of growth and evolution mechanism of martensite lath in CGHAZ of DP1180 steel: (**a**) MA1 nucleates at 50° with PAGB; (**b**) MA2 nucleates parallel to MA1; (**c**) MA3 nucleates in grains; (**d**,**e**) MA4 nucleates and grows at 90° with PAGB; (**f**) MA7 nucleates on PAGB; (**g**,**h**) MA9 nucleates on the side of MA8; (**i**) MA10 nucleates on carbide; (**j**) end of phase transition.

Seven typical growth modes of martensitic laths in the DP1180 steel CGHAZ during cooling are proposed as shown in Figure 7. The model diagram of the growth and evolution mechanism of martensite laths is shown in Figure 8.

The blue dotted line indicates the PAGB, while the red circle represents the carbide particles that precipitated on the grain boundary and within the grain. The obvious relief effect on the specimen’s surface is a result of the difference in specific volume between martensite and the original austenite during the gradual cooling process from 1347.8 °C to 492.7 °C, as shown in Figure 7a. When austenite transforms into martensite, its volume expands, resulting in martensite relief [17,22]. In addition, Yang et al. believed that this phenomenon was caused by martensitic shear [18]. Therefore, it can be determined that 492.7 °C is the starting temperature of martensitic transformation.

First, the new martensite lath, MA1, rapidly nucleates at an angle of 50° within the PAGB and finally penetrates the entire grain when the temperature decreases to 488.1 °C, as shown in Figure 7b. Second, the generated MA1 induces the nucleation of a new lath, MA2, in the crystal, which grows parallel to MA1 when the temperature drops to 466.4 °C in Figure 7c. The growth of MA2 ends after reaching the PAGB, until the temperature cools to 458.0 °C (see Figure 7d). Third, lath MA3 appears in the spontaneous nucleation of the crystal when the temperature is reduced to 452.0 °C, as seen in Figure 7e. However, the growth of MA3 ends due to an encounter with the grain boundary on one side during the growth process (see Figure 7f). Fourth, lath MA4, perpendicular to the grain boundary, appears when the temperature drops to 442.1 °C, as shown in Figure 7g. Lath MA4 bifurcates into two martensite laths, MA4_1_ and MA4_2_, during the growth process and grows in different directions (see Figure 7i). Finally, due to the collision of laths MA41 and MA42 with MA5 and MA6, respectively, the growth of MA4_1_ and MA4_2_ ends earlier, at 369.2 °C (see Figure 7m). Fifth, the width of lath MA7, with nucleation on the grain boundary, is 1.38 μm when the temperature is reduced to 436.2 °C, as seen in Figure 7h. With continuous cooling of the temperature to 375.6 °C, the width of lath MA7 increases to 2.36 μm and growth ends (see Figure 7l). Sixth, as shown in Figure 7i,j, the MA9 lath is attached to the side nucleation of the previously generated MA8 lath when the temperature decreases to 387.3 °C. Seventh, the martensite MA10 formed on the carbide particles appears when the temperature is reduced to 378.9 °C (see Figure 7k). When the temperature decreases, multiple martensite laths appear simultaneously in the whole martensitic transformation process (see the yellow marking in Figure 7n). The surface relief phenomenon vanishes upon cooling the sample to 291.3 °C, signifying the completion of martensitic transformation (see Figure 7o).

### 3.3. Evolution Characteristics of CGHAZ Microstructure

From the in situ observation results in Figure 7, it can be seen that the difficulty of nucleation is determined according to the order of martensite appearances when the CGHAZ of DP1180 steel undergoes martensitic transformation. Furthermore, the key factor determining the difficulty of martensitic lath nucleation is the energy barrier level. The lower the energy barrier, the easier the nucleation. Therefore, martensitic laths preferentially nucleate at positions with lower energy barriers. In addition to the surface energy formed by the new interface, strain energy is also introduced during martensitic transformation. Moreover, formation of the new phase mainly depends on elastic sliding of the interface, because the atoms cannot be rearranged by diffusion at low temperatures. Therefore, from the perspective of energy relationships, the kinetics of nucleation are expressed as follows (1):

Δ*G* = −Δ*G*_V_ + (Δ*G*_S_ + Δ*G*_E_) (1)

where Δ*G*_V_ is the volumetric free energy, Δ*G*_S_ is the surface energy, and Δ*G*_E_ is the elastic strain energy. The surface energy Δ*G*_S_ is relatively small, and the elastic strain energy Δ*G*_E_ becomes the main obstacle to martensitic transformation. The atoms are loosely arranged, and the gap is larger at the PAGB. The stress generated by the volume change during the growth of martensite can be released more easily, thereby reducing the strain energy and promoting the nucleation of martensite. Therefore, it is more conducive to the nucleation of martensite at the PAGB. This result is consistent with the literature [19] on the martensitic transformation behavior of 40Cr10Si2Mo steel by in situ observation technology. In addition, the martensite that nucleates at the grain boundary can induce the nucleation of new martensite within the crystal. This is attributed to the stress concentration resulting from the volume expansion effect [23,24].

In summary, during the martensitic transformation process, the martensitic lath progresses through three distinct stages: nucleation, growth, and cessation of growth. The priority of martensite lath nucleation positions is obtained according to the energy barrier (see Figure 8). The order from easy to difficult is as follows: 50° angle nucleation with the PAGB, intragranular nucleation of a new lath induced by a formed lath, intragranular spontaneous nucleation, 90° angle nucleation with the PAGB, nucleation on the PAGB, nucleation on the side of the generated lath, and carbide particle nucleation. Finally, most of the laths stop growing when they collide with grain boundaries or other laths, thus forming complex interlocking structures.

### 3.4. Martensitic Lath Growth Rate

In this study, the martensite lath nucleation growth rate during cooling is quantitatively analyzed using Image-Pro Plus software.

The relationship between the growth rate of martensite laths and temperature can be expressed as

**Figure 9:**
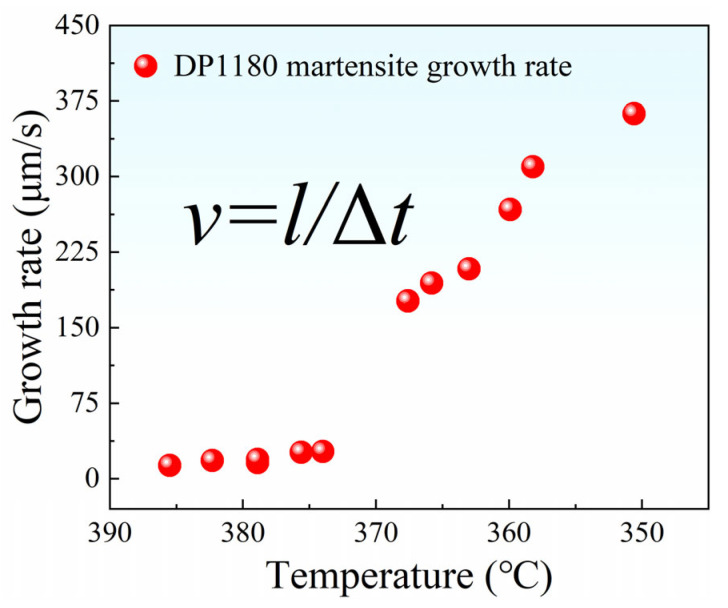
Temperature dependence of the growth rate of martensitic laths in the coarse-grained zone of DP1180 steel.

*v* = *l*/Δ*t* (2)

where *l* is the length of the lath, and Δ*t* is the time used for the growth of the laths. The growth rate of martensite laths is calculated to be between 13.2~362.32 μm/s. The data presented reveals that the growth rate of the lath will increase significantly with the temperature decrease when the CGHAZ of DP1180 steel undergoes martensitic transformation, as shown in Figure 9. In this process, the nucleation order of the laths is mainly determined by the energy barrier, and the growth rate is dominated by temperature change. We assume that the prior austenite grain size, cooling rate, and other factors remain constant and unaffected by the change in phase transition temperature. The starting temperature of martensitic transformation is systematically calculated and analyzed according to the theoretical model proposed by Andrews [25], and the calculation formula is

*M_S_* = 539 − 423*ω*_C_ − 12.1*ω*_Cr_ − 7.5*ω*_Mo_ − 30.4*ω*_Mn_ − 17.7*ω*_Ni_ (3)

where *M_S_* is the martensitic phase transition starting temperature (°C), and *ω*_M_ is the mass fraction (%) of the alloying element *M* (*M* = C, Cr, Mo, Mn, Ni) in DP1180 steel. The martensitic transformation starting temperature of DP1180 steel is calculated to be *M_S_* = 417.86 °C.

The temperature at which a martensitic lath *i* begins to grow is defined as *T_i_* during in situ observation. Subsequently, the supercooling Δ*T* during the growth of the lath is calculated according to the formula Δ*T_i_* = *M_S_* − *T_i_* [18], where *M_S_* is the onset temperature of martensitic phase transition. The supercooling of the corresponding martensitic lath is calculated and combined with Figure 5; the specific values are shown in Table 3.

From Table 3, it can be seen that Δ*T* increases with the decrease in temperature, which makes the growth rate of martensite laths increase as a whole. However, the growth behavior of some laths is inconsistent with this trend. This may be affected by factors such as irregular atomic arrangements, lattice distortions, or defects [26,27,28]. Therefore, the growth rate of the laths presents an upward trend with the decrease in temperature. The main reason for this phenomenon is the increase in material undercooling. The greater the undercooling, the faster the growth rate of the lath.

### 3.5. EBSD Characterization of CGHAZ Microstructure

**Figure 10:**
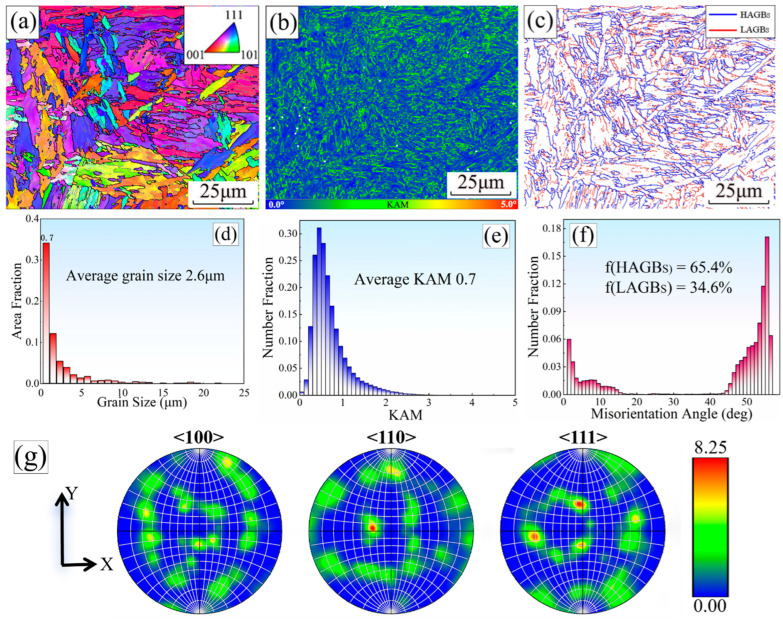
EBSD characterization of DP1180 steel CGHAZ: (**a**) IPF, (**b**) KAM, (**c**) distribution of grain boundaries, (**d**) grain size statistical chart, (**e**) KAM statistical chart, (**f**) histogram of angular distribution of vectorial difference, and (**g**) pole figure.

EBSD characterization is carried out on the samples after in situ observation with the LSCM, as shown in Figure 10. The inverse pole figure (IPF) is the projection distribution of the corresponding appearance direction of each grain in the crystallographic orientation coordinate system. The difference in grain color represents the difference in grain orientation, and the single or diversified color represents the difference in grain orientation in Figure 10a [29,30]. The martensite laths in the original austenite grains grow in different directions. The crystallographic characteristics of martensitic laths are almost the same in the same grain, but they are far apart in space. These laths may be the same variant pairs in the Kurdjumov–Sachs (K-S) orientation relationship [31].

The strain gradient is blue → green → yellow → red in the kernel average misorientation (KAM) of adjacent grains, as shown in Figure 10b. Among them, the low-strain zone is represented as blue, and the high-strain zone is represented as red. The edge of the lath and some of the internal areas of the lath are green and show remarkable strain concentration, indicating that the strain energy in these areas is high and evenly distributed. At the same time, the KAM graph can directly represent the distribution of the geometrically necessary dislocation (GND) density, and the higher the KAM, the higher the density of GND [31,32,33]. A KAM value of 0.7° and a GND value of 5.83 × 10^14^/m^2^ can be obtained for this region by using the AztecCrystal 2.1 EBSD software. Figure 10e demonstrates low inhomogeneity in crystal orientation, indicating a small difference in orientation within this region.

According to the literature [20], an orientation difference angle between the neighboring grains in the range of 2° to 15° is defined as low-angle grain boundaries (LAGBs), and an orientation difference angle between the neighboring grains greater than 15° is defined as high-angle grain boundaries (HAGBs). In the grain boundary distribution map, LAGBs are represented by red solid lines, and HAGBs are represented by blue solid lines (see Figure 10c). The volume fractions of high-angle grain boundaries and low-angle grain boundaries are 60.5% and 39.5%, respectively (see Figure 10f). The obtained CGHAZ structure is dominated by high-angle grain boundaries, which are distributed at the martensite lath boundaries. The low-angle grain boundaries are distributed inside the martensite lath. This is because the formation of high-angle grain boundaries reduces the total interface energy of the system through grain boundary migration and rearrangement during the phase transition process. Low-angle grain boundaries are mostly formed by dislocation accumulation and rearrangement, which are common in martensitic laths. The local stress field inside the martensite lath is small, which is not enough to drive the formation of high-angle grain boundaries.

The grain sizes in this region have a maximum of 21.9 μm, a minimum of 0.7 μm, and an average of 2.6 μm, with a standard deviation of 2.9 μm, as shown in Figure 10d. This result illustrates that the grain uniformity in this area is poor, the size distribution is wide, and the change is large. This may be because grain growth will be affected by the mutual competition of surrounding grains during the phase transition process. Some of the grains may take advantage during growth, making their size larger. At the same time, the growth of other grains may be inhibited, resulting in smaller sizes. Finally, this competition mechanism results in the inhomogeneity of grain size distribution. The distribution of grain sizes is observed by counting the frequency (i.e., the number of occurrences) of different grain sizes. The highest frequency of occurrence is 0.7 μm (341 times). The lowest frequency is 6.6 μm (6 times). The angle distribution histogram of adjacent grain orientation differences shows bimodal distribution characteristics.

The peak values appear at low angles (2° < θ < 5°) and high angles (54° < θ < 56°) in Figure 10f. In addition, some studies have confirmed that the bimodal grain boundary distribution is more conducive to metal materials with high strength and good ductility [34]. It can be observed from the pole figure, Figure 10g, that different crystal orientations are randomly distributed. There are red high-density areas in the <110> and <111> directions, while there is no red high-density area in the <100> direction. Therefore, the peak strength in the <110> and <111> directions is higher than that in the <100> direction, with the maximum density reaching 8.25.

## 4. Conclusions

In this work, the phase transformation behavior, growth rate, and nucleation growth process of martensite in the CGHAZ of dual-phase steel DP1180 were examined under simulated laser welding conditions by in situ observation. Several important conclusions are summarized as follows:

(1) The initial temperature of martensitic transformation was determined to be 492.7 °C according to the relief effect. Multiple martensite laths began to appear at the same time as the temperature decreased. The martensitic transformation ended at 291.3 °C.
(2) Seven growth modes of CGHAZ martensitic laths are put forward: 50° angle nucleation with the PAGB, intragranular nucleation of a new lath induced by a formed lath, intragranular spontaneous nucleation, 90° angle nucleation with the PAGB, nucleation on the PAGB, nucleation on the side of the generated lath, and carbide particle nucleation.
(3) The internal strain energy of the edge of the lath and part of the internal area of the lath is high and evenly distributed. The orientation difference between adjacent grains primarily involves high-angle grain boundaries. The grain size distribution in the CGHAZ is not uniform.

## Figures and Tables

**Table 1:** Chemical composition of DP1180 steel (wt.%).

Material	C	Si	Mn	P	S	Alt	Ti	Fe
DP1180	0.1139	0.215	2.4	0.01	0.0045	0.045	0.0185	Bal.

**Table 2:** Basic mechanical properties of DP1180 steel.

Material	Tensile Strength/MPa	Yield Strength/MPa	Elongation/%
DP1180	1230–1300	1000–1106	9–10

**Table 3:** Calculated values of supercooling and growth rates for martensitic laths in DP1180 steel.

Martensite Lath (*i*)	Δ*T*/°C	*v*/(μm·s^−1^)
1	32.4	13.2
2	35.6	17.9
3	39	15.8
4	39	19.1
5	42.3	26.2
6	43.9	26.9
7	50.3	176.5
8	52.1	194
9	54.9	208.2
10	58	267.3
11	60	309.5
12	67.3	362.3

## Data Availability

The data presented in this study are available on request from the corresponding author due to the raw/processed data required to reproduce these findings are part of the ongoing research.
